# The Presence of Sulphocyanegen in Human Saliva

**Published:** 1846-09

**Authors:** 


***The Presence of Sulphocyanogen in Human Saliva.***—M. Petenkofer observes in the ***London Chemical Gazette***, in reference to this subject, that as authors are not agreed upon the occurrence of sulphocyanogen in the saliva,

Gmelin, Ure, Liebig and Wright, speaking in favor of it, whilst Berzelius, Kuhn and Muller are opposed to it, it appears requisite to the author again to investigate the subject. The saliva used was collected from the author himself, and its secretion was promoted by smoking tobacco. It was acidified with sulphuric acid, and subjected to distillation to separate the volatile acids. After twenty-four grammes of the fluid had been distilled to about two-thirds, the author observed that when carbonate of lead was put into the receiver, it was blackened by sulphuretted hydrogen. This was supposed to arise from the presence of a metallic sulphuret in the saliva; but none could be detected; moreover, it was shown by another experiment, that sulphocyanide of potassium gave off sulphuretted hydrogen when distilled with sulphuric acid. The carbonate of lead used in the above distillation was boiled with water, and the filtrate evaporated to dryness. The residue was now boiled with alcohol, and the alcoholic extract also evaporated to dryness. On adding sulphuric acid to it, the odor of acetic acid was distinctly evolved. The portion of the aqueous extract insoluble in alcohol was composed of chloride of lead; hence muriatic and acetic acids had passed over. To separate the constituents of the portion insoluble in water, it was treated with a weak solution of carbonate of soda, the filtrate evaporated and exhausted with spirit. The spirituous solution was reddened by chloride of iron. The reddening certainly was less intense than that of the ordinary spirituous extract of saliva; still even this experiment goes far towards proving the presence of sulphocyanogen. The author next determined to examine carefully the means for detecting sulphocyanic acid. It is well known that acetic, formic and meconic acids produce the same reactions with chloride of iron as sulphocyanic acid. It is also known that a solution of peracetate of iron becomes turbid when boiled, but becomes nearly clear again on cooling. The latter phenomenon also occurs when sulphate of potash, nitre, chloride of sodium, &c., are heated with a solution of acetate of iron, and as the author has observed, by continued ebullition with a sufficient quantity of chloride of sodium or potassium, the whole of the iron is precipitated; so that on cooling it requires a very considerable quantity of acetic acid to redissolve it. If the reddish-brown precipitate be collected on a filter, at first a transparent fluid passes through, containing no trace of iron; but after continued edulcoration, the precipitate on the filter begins to dissolve. Peroxide of iron and chlorine are detected in the solution; and if a little nitric acid be added to it, the greater part of the iron sides in bright brown flakes. It has a powerful astringent, but not the least inky taste; and if metallic chlorides be added to it, the peroxide of iron it contains is again precipitated. The above dark brown solution is undoubtedly the basic chloride of iron mentioned by Phillips.

To examine this compound more minutely, the author burnt piano-forte wire in a current of chlorine, and digested the aqueous solution of the chloride thus obtained with hydrated peroxide of iron until the acid reaction and inky taste had disappeared. On estimating the chlorine by silver and precipitating the iron by ammonia, the author obtained the formula $2Fe^3Cl+2Fe^2O^3$. The solution became turbid on evaporation, and hydrated peroxide of iron separated.

Thus, the property of fluids which are of a red color from the presence of acetate of iron, of being perfectly decolorised on ebullition with alkaline metallic chlorides, enables us to distinguish them readily from the precipitation produced by sulphocyanic acid. Formic acid reacts upon peroxide of iron exactly as acetic acid, except that the precipitation of the iron on boiling occurs much sooner, especially when chlorides are added. The red color caused by meconate of iron cannot be removed by this means. To distinguish the sulphocyanate of iron from the meconate and the acetate, the author recommends the red ferrocyanide of potassium. Thus, if the red ferrocyanide of potassium be added to the red solution of chloride of iron and sulphocyanide of potassium, Prussian blue falls in a short time, and immediately if heat be applied. The odor of prussic acid also becomes evident. An explanation of this process cannot at present be given with certainty; it may, however, be remarked, that no products of the decomposition of sulphocyanogen can be detected, but that the greenish fluid again yields Prussian blue on the addition of chloride of iron and red ferrocyanide of potassium. The latter salt does not exert the least action upon the meconate, acetate, &c., of iron.

To apply these results to test the saliva, the author evaporated fresh saliva almost to dryness, exhausted it with strong spirit, again evaporated, and dissolved the alcoholic residue in water. The solution was very strongly reddened by neutral chloride of iron, and let fall some brown flakes; but it could not be caused to disappear by the addition of chloride of sodium nor of ammonium. A very minute portion only of the color could, therefore, have been caused by acetates. Another portion of the salivary extract was reddened by a few drops of chloride of iron, and treated with red ferrocyanide of potassium, whereupon the formation of Prussian blue described above occurred. Another portion of the extract was boiled with chlorate of potash, muriatic acid being at the same time added in drops, chloride of barium then gave a precipitate of sulphate of baryta, which was not the case previously. When the extract was treated with a solution of lead in caustic potash, no formation of sulphuret of lead could be detected; but when it was boiled with sulphuric acid, and a moist piece of lead-paper held over it, it was distinctly rendered brown. By numerous repetitions of the above experiments on the saliva of different persons, the author obtained the same results; so that the occurrence of a metallic sulphocyanide in saliva may be admitted with certainty.

To estimate the sulphocyanogen in saliva, Wright advises us to form sulphocyanide of lead, to weigh this, and to calculate the quantity of sulphocyanide of potassium, in the saliva from it. But the metallic chlorides, &c., in the saliva inevitably produce error, consequently, according to Wright's experiments, the amount of sulphocyanogen in the saliva is much too great. Hence the author recommends the oxidation of the sulphocyanogen, so as to form sulphuric acid, and the estimation of the latter by baryta. A weighed quantity of saliva is evaporated, exhausted with alcohol, the latter evaporated, the residue dissolved in water and boiled, chlorate of potash and a little muriatic acid being added. Before adding the muriatic acid, the fluid must be heated to boiling, otherwise, a little sulphocyanic acid will escape undecomposed. The author thinks that the small quantity obtained by Kuhn, may be explained in this manner. 100 parts of sulphate of baryta correspond to 25.11 sulphocyanogen, or 41.91 sulphocyanide of potassium. In this analysis care must be taken that no other sulphur-compound exists in the solution. The alcoholic solution of the solid residue of saliva leaves almost all the sulphates which may be present undissolved. If not, their separation must be previously effected by a soluble salt of baryta; sulphurous acid and hyposulphuric acids must be previously converted into sulphuric acid by peroxide of lead. Metallic sulphurets might be removed by digestion with carbonate of lead. The author ascertained, by counter experiments, that the sulphur of the sulphocyanogen is completely oxidized in the above manner.

The mode of formation of the sulphocyanogen in saliva has hitherto proved a stumbling block; but if we regard urea as a cyanate of ammonia, $C_2NO + NA^4O$, and compare the formula of sulphocyanide of ammonium with it, $C_2NS_2 + NH_3$, we find a striking relation between them; for if we replace 2 equiv. of oxygen in the urea by 2 equiv. of sulphur, we have the elements of sulphocyanide of ammonium, $C_2N_2H_4S_2 = C_2NS_2 + NH_4$. Moreover, the products of decomposition of the two substances on destructive distillation are to a certain extent similar. The author has also found that urea may be converted into a compound of sulphocyanogen by the action of alkaline sulphurets. Since urea occurs already formed in the blood, it would not appear improbable that by combining in the salivary glands with the sulphur of the protein compounds, it forms sulphocyanogen. Wright has remarked the excretion of urea in the saliva during salivation. It would be interesting to observe whether saliva contains urea in mercurial ptyalism where the chloride of iron frequently does not cause any reddening.

